# A Dual-Polarized Omnidirectional Rectenna Array for RF Energy Harvesting

**DOI:** 10.3390/mi14051071

**Published:** 2023-05-18

**Authors:** Yong Wang, Ningning Lu, Hucheng Sun, Rui Ren

**Affiliations:** 1 Jiangsu Collaborative Innovation Center of Atmospheric Environment and Equipment Technology, Nanjing University of Information Science and Technology, Nanjing 210044, China; 2 Beijing R&D Center, The 54th Research Institute, China Electronics Technology Group Corporation, Beijing 100041, China; 3 College of Science, Hohai University, Nanjing 210044, China

**Keywords:** RF energy harvesting, dual-polarized, omnidirectional antenna, rectenna

## Abstract

In this paper, a dual-polarized omnidirectional rectenna array using a hybrid power-combining scheme is proposed for the applications of RF energy harvesting. In the antenna design part, two omnidirectional antenna subarrays are created to receive horizontally polarized electromagnetic (EM) waves and a four-dipole subarray is produced to receive vertically polarized incoming EM waves. The two antenna subarrays of different polarizations are combined and optimized, so as to reduce the mutual influence between them. In this way, a dual-polarized omnidirectional antenna array is realized. In the rectifier design part, a half-wave rectifying structure is adopted for converting the RF energy into DC energy. Based on the Wilkinson power divider and 3-dB hybrid coupler structure, a power-combining network is designed to connect the whole antenna array and rectifiers. The proposed rectenna array is fabricated and measured under different RF energy harvesting scenarios. All simulated and measured results are in good agreement, which verifies the capabilities of the designed rectenna array.

## 1. Introduction

RF energy harvesting (REH) is the technology by which energy is derived from sources in the ambience, and then stored for use by low-power electronics [1]. In recent years, the demand for low-power electronics in smart homes [2], smart healthcare [3], and environmental monitoring [4] has increased substantially. The energy consumption of these devices has attracted widespread attention. REH has become a suitable choice for these low-power electronics [5,6]. Compared with other types of energy in the ambience, such as solar energy, wind energy, etc., the ubiquitous ambient RF energy has its unique advantages [7]. First, there is a wide range of RF energy sources [8]. Ambient RF energy can be obtained from the ubiquitous wireless transmitters around, including cell phones, handheld radios, mobile base stations, TV/radio stations, wireless routers, etc. On the other hand, RF energy sources are stable. For example, TV broadcast signals can be collected by energy harvesting systems throughout the day without having to consider energy interruptions. Therefore, the use of this free and inexhaustible ambient RF energy is a promising topic, and a lot of research work [9,10,11,12,13,14] is devoted to RF energy harvesting.

In a REH system, the key device is the rectenna which harvests the incident electromagnetic (EM) wave and converts RF power into DC power. The power conversion efficiency (PCE) is the main concern of the rectenna’s performance, which is defined as the ratio of the output DC power to the input RF power. Thus, many research efforts have been dedicated to improving the PCE of rectennas in different application scenarios [15,16]. In the process of REH, the direction of the incident EM wave in the ambience is unknown, which could change at different moments [17,18,19,20]. Therefore, to collect EM waves from different directions, an omnidirectional antenna can be used to cover a larger area [21,22]. In addition, the diverse sources of EM waves in the ambience lead to unknown polarizations of the incident EM waves. If a linearly polarized antenna is used for REH, the polarization mismatch problem may be encountered, which reduces the performance of the system. A dual-polarized antenna can be considered for REH, decomposing the incoming wave of any polarization into two components of vertical and horizontal polarizations [23,24,25]. The RF energy of these two polarization components can be received separately and then energy conversion is performed, thus avoiding polarization loss. In addition, to collect as much energy as possible, antenna arrays can be used to receive RF energy with more antenna elements. For rectennas based on antenna arrays, RF power combining or DC power combining can generally be utilized. However, for ambient REH, because the directions and power levels of incoming EM waves are time-varying, merely using RF power combining or merely using DC power combining is not the optimal choice [26,27]. A combination of the two methods can be considered according to the application scenarios. The above ideas are fully considered and applied to this research work.

In this paper, a dual-polarized omnidirectional rectenna array with a hybrid power-combining scheme is proposed for REH applications. An omnidirectional antenna array containing horizontally and vertically polarized subarrays is designed for RF power receiving. Then, a hybrid power-combining network is created to evenly distribute all the received RF power into two portions. By merging the antenna array, power combining network, and designed rectifiers, a rectenna array is formed. Measurement results show that the rectenna array achieves good omnidirectional-recycling capability. In addition, it can maintain excellent stability when receiving EM waves at different incidence angles. Therefore, the proposed rectenna array is ready for applications in REH.

## 2. Rectenna Array Design

### 2.1. Antenna Design

**Figure 1:**
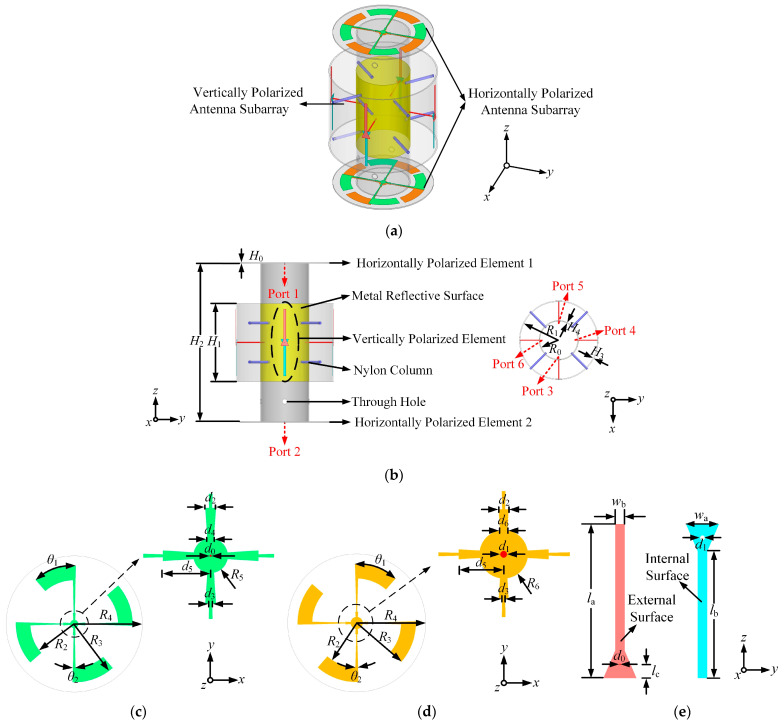
Structure of the antenna array: (**a**) 3D view; (**b**) Overall layout; (**c**) Top view of the horizontally polarized element; (**d**) Bottom view of the horizontally polarized element; (**e**) Zoomed-in view of the vertically polarized element.

A dual-polarized omnidirectional antenna array operating at 2.45 GHz is proposed, which comprises a horizontally polarized array and a vertically polarized array. The structure of the dual-polarized omnidirectional antenna array is shown in Figure 1. The horizontally polarized antenna subarray is designed with a double-layer structure to improve the gain, which contains two identical horizontally polarized omnidirectional elements, 1 and 2. The green and orange parts in Figure 1c,d represent the metal layers printed on the top and bottom of the horizontally polarized element, respectively. Each of the metal layers is printed on a 30-mil-thick Rogers RO4350B substrate with a dielectric constant of 3.66 and a loss tangent of 0.0037. The distance between the two horizontally polarized elements 1 and 2 is approximately one wavelength. From Figure 1c,d, each curved radiating branch of the horizontally polarized element is connected to the tapered balun by a tapering microstrip line and converges at the feed point. The location of the feed point is at the center of the circular dielectric substrate.

As can be seen from Figure 1a,b, the vertically polarized antenna subarray consists of four identical dipole elements, and the design introduces a gradient balun structure to improve the impedance matching of the antenna elements. This subarray contains two inner and outer layers of C-UV 9400E cylindrical dielectric substrates of different heights. The dielectric constant and loss tangent of the C-UV 9400E substrate are 3 and 0.05, respectively. The thicknesses of both cylindrical dielectric substrates are 0.8 mm. The yellow part in Figure 1a,b is a metal reflector printed on the inner cylindrical dielectric substrate. The dipole elements are co-formed on both sides of the outer cylindrical dielectric substrate. The inner and outer cylindrical dielectric substrates are connected and fixed by eight nylon columns to improve the structural stability of the antenna array. The two circular dielectric substrates and the inner and outer cylindrical dielectric substrates are placed concentrically in the layout. The inner cylinder dielectric substrate is placed between two circular substrates and fits closely to the bottom of the upper dielectric substrate and the top of the lower dielectric substrate, respectively. Each part of the antenna array was simulated and optimized using the software Ansys HFSS. The optimized dimensions of the proposed antenna array are given in Table 1.

**Figure 2:**
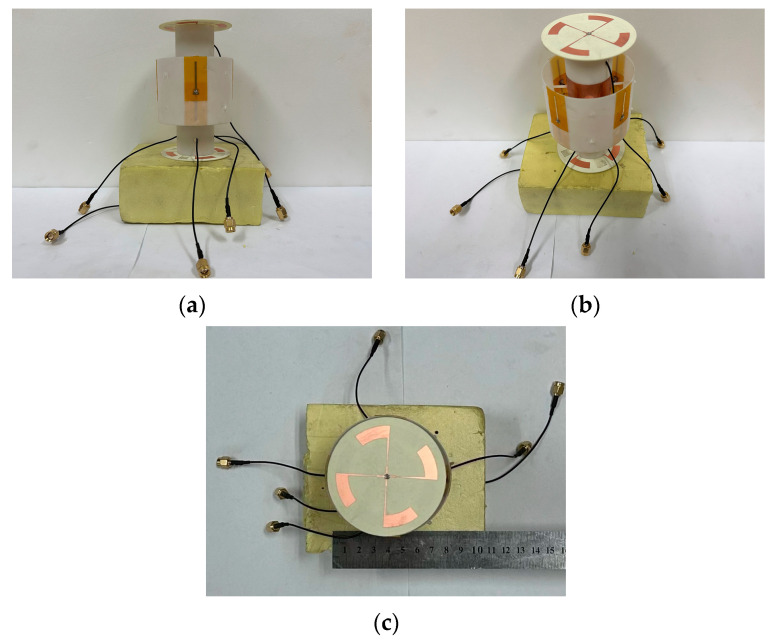
Photographs of the fabricated antenna array: (**a**) Side view; (**b**) Oblique view; (**c**) Top view.

To verify the performance of the design, the antenna array was fabricated and measured. Figure 2 provides photographs of the fabricated array. Due to the limited internal space of the inner cylindrical dielectric substrate, it is difficult to use conventional SMA connectors for soldering. Thus, 50-Ω SMA connectors with RF cable wires are used for power feeding to each element of the antenna array. The length of each RF cable is 0.2 m, and its measured insertion loss at 2.45 GHz is 0.26 dB. The inner and outer cylindrical dielectric substrates are fabricated using 3D printing technology. Considering the fabrication cost and complexity, the vertically polarized elements are printed on 45-μm-thick flexible polyimide films with a dielectric constant of 3.5 and a loss tangent of 0.008. Then, the fabricated films are laminated to the surface of the outer cylindrical dielectric substrate to achieve conformality. During assembly, the corresponding through-holes of the inner and outer cylindrical substrates are aligned and fixed by nylon columns. In addition, the relative positions of the upper and lower horizontally polarized elements should be precisely aligned.

**Figure 3:**
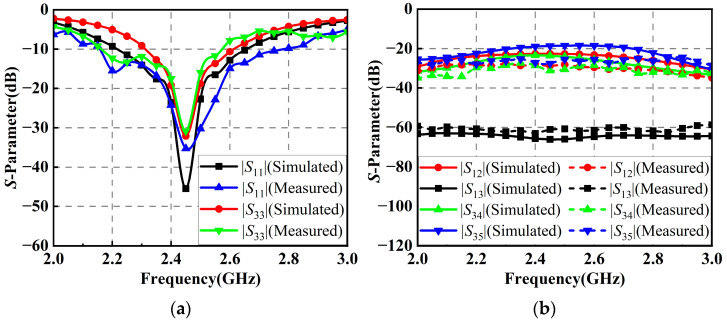
*S*-parameters of the elements in the proposed antenna array: (**a**) Reflection coefficients; (**b**) Isolation performances.

Figure 3a shows the reflection coefficients of the horizontally polarized element 1 and the vertically polarized element 3 in the antenna array. It can be seen that the measured results are in good agreement with the simulated ones. The measured *S*-parameters of the horizontally polarized element 1 are below −10 dB in the range of 2.16 GHz to 2.77 GHz, corresponding to a percentage bandwidth of 24.7%, while the measured *S*-parameters of the vertically polarized element 3 are below −10 dB in the range of 2.17 to 2.58 GHz, corresponding to a percentage bandwidth of 17.3%. At the center frequency of 2.45 GHz, the *S*-parameters of both horizontally and vertically polarized elements are below −30 dB. Due to the symmetry, as the performances of those elements with the same polarization are almost identical, the *S*-parameters of the rest of the elements are not shown. Figure 3b gives the isolation performances between the antenna elements in the antenna array. Within the operating bandwidth of the antenna array, the measured isolations between the elements with the same polarization are better than 20 dB, while the isolations between those elements with different polarizations are better than 55 dB.

**Figure 4:**
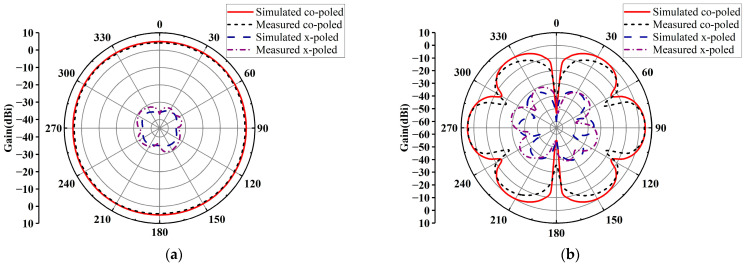
Radiation patterns of the antenna array at 2.45 GHz in the horizontal polarization: (**a**) xoy plane; (**b**) yoz plane.

**Figure 5:**
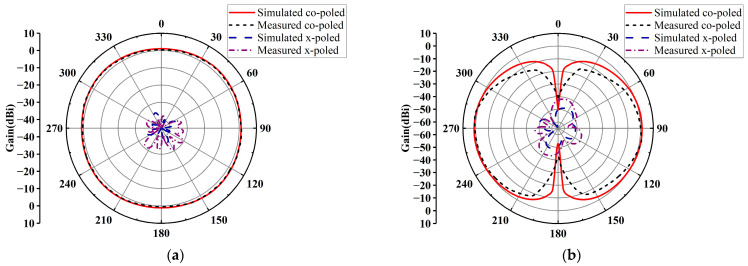
Radiation patterns of the antenna array at 2.45 GHz in the vertical polarization: (**a**) xoy plane; (**b**) yoz plane.

The radiation patterns of this antenna array were measured in an anechoic chamber. Figure 4 depicts the radiation patterns of the antenna array in the xoy and yoz planes at 2.45 GHz in the horizontal polarization. The measured results show that in the horizontal xoy plane, the antenna array has good omnidirectional radiation performances with a gain fluctuation of 0.85 dB and a peak gain of 5.01 dBi. In both xoy and yoz planes, the cross-polarizations of the antenna array are below −30 dB. Figure 5 plots the radiation patterns of the antenna array in the xoy and yoz plane at 2.45 GHz in the vertical polarization. The measured results show that in the horizontal xoy plane, the peak gain of the antenna array is 1.83 dBi and the gain fluctuation is 1.36 dB. In the xoy plane, the cross-polarization of the antenna array is below −30 dB, while the cross-polarization in the yoz plane is below −40 dB. The measured peak gains are slightly lower than the simulated ones, but within the allowable margin of error, probably due to errors in the fabrication and assembly of the antenna array. From the above results, the antenna array shows good omnidirectional performances in both horizontally and vertically polarized operating states and can be readily applied for RF energy harvesting.

### 2.2. Rectifier Design

**Figure 6:**
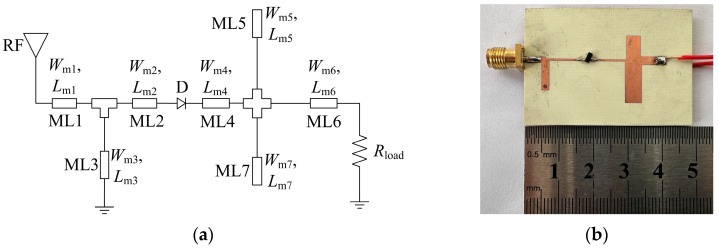
Schematic and photograph of the proposed rectifier: (**a**) Schematic; (**b**) Photograph.

A rectifier operating at 2.45 GHz is designed and optimized using the Advanced Design System (ADS) software, which is printed on a 30-mil-thick Rogers RO4350B substrate with a dielectric constant of 3.66 and a loss tangent of 0.0037. Its circuit schematic is shown in Figure 6a. The key parameters of the Schottky diode HSMS-2850 are breakdown voltage (B_V_), threshold voltage (V_T_), and junction capacitance (C_J0_), which are 3.8 V, 0.35 V, and 0.18 pF, respectively. Compared with other commonly used RF diodes, the Schottky diode HSMS-2850 has a relatively small threshold voltage. Thus, it is advantageous to rectify low RF power and is suitable for REH applications. Furthermore, its junction capacitance supports its operation at 2.45 GHz. Therefore, the Schottky diode HSMS-2850 is used in this design.

**Figure 7:**
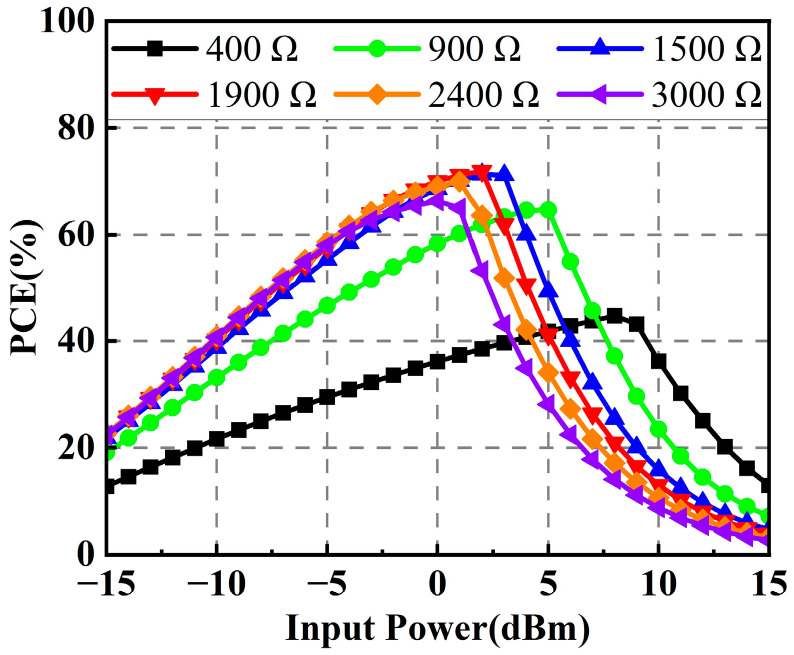
Simulated PCEs of the rectifier under different load resistances.

After fabrication, the photograph of the rectifier is given in Figure 6b. The optimized dimensional parameters of the proposed rectifier are listed in Table 2. Figure 7 shows the simulated PCEs of the rectifier under different load resistances. It is obvious that the operating input power range for PCE larger than 50% becomes narrow when the load resistance goes beyond 1900 Ω. On the other hand, when the load resistance gets smaller than 1900 Ω, the maximum PCE decreases sharply. Therefore, considering the operating input power range and the maximum PCE, a load resistance of 1900 Ω is chosen as the optimal value in this design.

**Figure 8:**
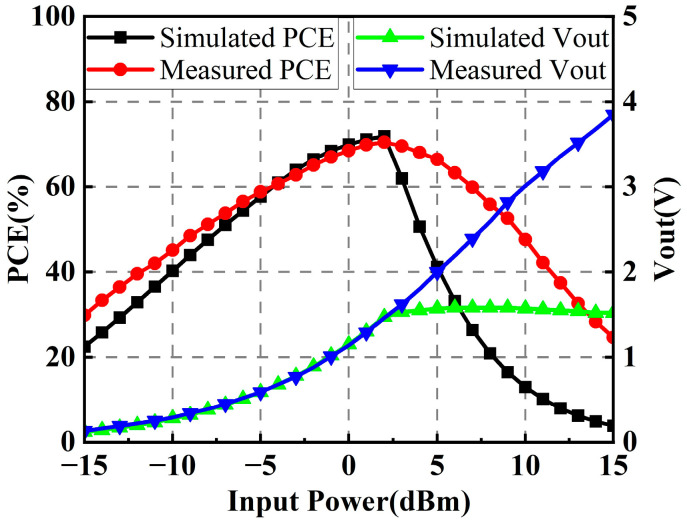
Simulated and measured PCEs and output voltages of the rectifier.

Figure 8 plots the simulated and measured PCEs and output voltages of the designed rectifier. It can be seen that the fabricated rectifier arrives at a maximum measured PCE of 70.5% when the input RF power is 2 dBm, and the corresponding output DC voltage is 1.46 V. Meanwhile, the measured PCE is higher than 50% over the input power range of approximately −8 dBm to 9 dBm. In the following dual-polarized rectenna array, two identical rectifiers designed here will be arranged in parallel as a rectifier array.

## 3. Power-Combining Scheme for the Rectenna Array

In the design of rectenna arrays, two possible power-combining schemes can be considered, i.e., RF power combining and DC power combining. RF power combining is to first combine the RF power received by the array elements, and then convert the total RF power to DC power. On the other hand, DC power combining is to first rectify the RF power outputs of the array elements into DC power outputs, and then combine them together.

During the RF energy harvesting, there are large discrepancies between the power levels received by the six ports of the proposed antenna array, which is detrimental to the PCE of the whole system. In addition, as the incident direction of the EM wave varies, the ratios of those received power levels will change as well. As discussed in [26,27], when the input power ratio of the two parallel rectifiers is changed, the PCE of the rectifier array will also be degraded. Therefore, under this circumstance, just using RF power combining or just using DC power combining is not the best choice. In this design, the horizontally polarized RF power and the vertically polarized RF power are combined separately into two independent portions. The RF power from all the horizontally polarized EM waves is combined into one port and named the horizontally polarized port (H-port). Similarly, the RF power from all the vertically polarized EM waves is combined into another port and named the vertically polarized port (V-port). Then, the two portions of RF power are evenly distributed by a 3-dB hybrid coupler, which can help the backend rectifiers obtain a more stable PCE.

### 3.1. RF Power Distributing Mechanism

**Figure 9:**
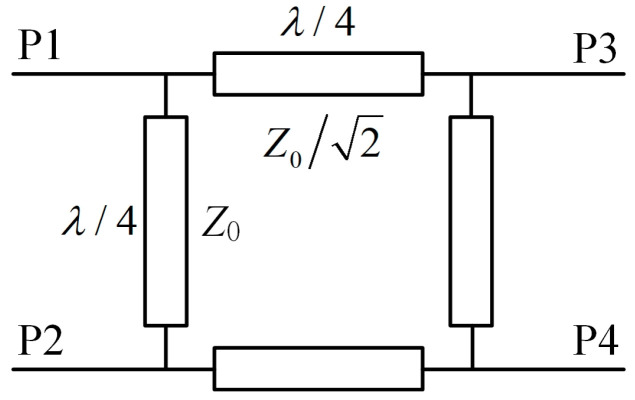
Schematic diagram of a 3-dB hybrid coupler.

Figure 9 shows the schematic of a 3-dB hybrid coupler. Using the transmission line theory, the coupler’s *S*-parameter matrix is derived and given in (1) below.

(1) $$S_{ij}=\frac{-1}{\sqrt{2}}\left(\begin{array}{cccc} 0 & 0 & j & 1\\ 0 & 0 & 1 & j\\ j & 1 & 0 & 0\\ 1 & j & 0 & 0 \end{array}\right)$$

where *S_ij_* is the transmission coefficient between the ports *P_i_* and *P_j_* of the hybrid coupler.

If its input ports P1 and P2 are connected to the combined H-port and V-port of the antenna array respectively, then the voltages of the output ports P3 and P4 can be calculated from the *S*-parameter:

(2) $$V_{P3}=V_{HP}\cdot S_{13}+V_{VP}\cdot S_{23}=\frac{-1}{\sqrt{2}}(jV_{HP}+V_{VP})$$

(3) $$V_{P4}=V_{HP}\cdot S_{14}+V_{VP}\cdot S_{24}=\frac{-1}{\sqrt{2}}(V_{HP}+jV_{VP})$$

where *V_HP_* and *V_VP_* denote the total voltage of horizontally polarized power and vertically polarized power, respectively. Hence, the power outputs at ports P3 and P4 are

(4) $$P_{P3}=\frac{{\left|V_{3}\right|}^{2}}{2Z_{0}}=\frac{V_{HP}^{2}+V_{VP}^{2}}{4Z_{0}}$$

(5) $$P_{P4}=\frac{{\left|V_{4}\right|}^{2}}{2Z_{0}}=\frac{V_{HP}^{2}+V_{VP}^{2}}{4Z_{0}}=P_{P3}$$

where *Z*_0_ is the characteristic impedance of the feeding lines at the coupler’s ports. According to (4) and (5), it can be found that the RF power output at port P3 and port P4 are equal. Thus, using two rectifiers in parallel to rectify the two portions of equal RF power, a stable PCE can be achieved.

### 3.2. Design of an RF Power-Combining Network

**Figure 10:**
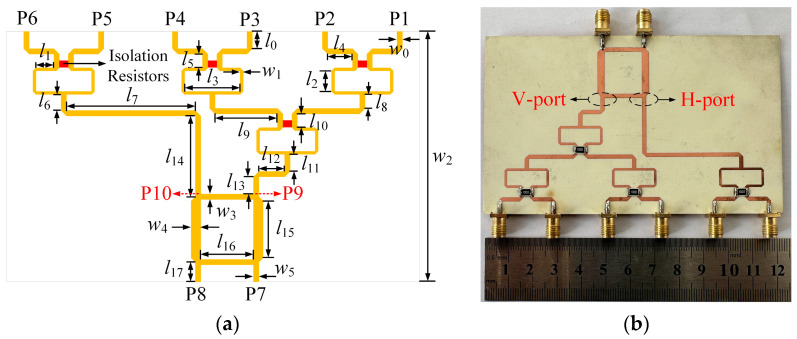
Schematic and photograph of the proposed RF power-combining network: (**a**) Schematic; (**b**) Photograph.

Based on the aforementioned mechanism, an RF power-combining network is proposed to integrate the RF power components of different polarizations received by the dual-polarized antenna array. Figure 10a shows the structure of the proposed RF power-combining network. The yellow parts in Figure 10a are all metal layers, and the red parts indicate isolation resistors with a resistance of 100 Ω. The RF power-combining network consists of a 1-to-2 Wilkinson power divider, a 1-to-4 Wilkinson power divider, and a 3-dB hybrid coupler. After optimization, the network was fabricated and measured, and a photograph is provided in Figure 10b. It is printed on a 30-mil-thick Rogers RO4350B substrate. Table 3 gives the detailed dimensions of the RF power-combining network.

**Figure 11:**
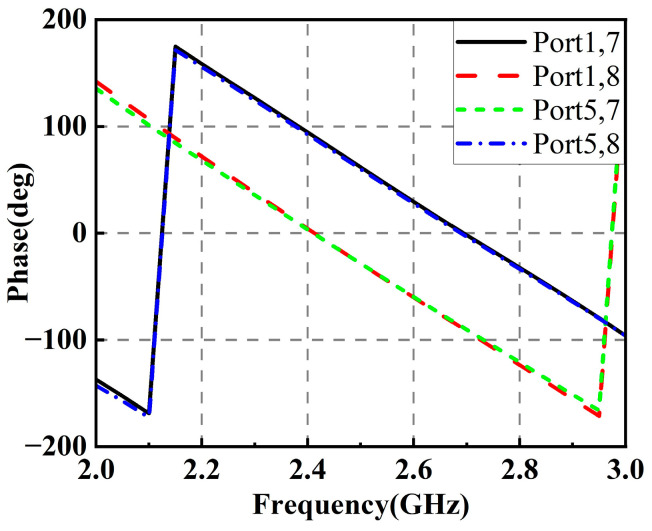
Phase simulation results of the proposed RF power-combining network.

Figure 11 plots the phase simulation results for the corresponding ports of the RF power-combining network. It can be seen that the network has an equal phase difference between the two Wilkinson power dividers’ input ports and the coupler’s output ports. In addition, there is a phase difference of about 90 degrees between either input ports and the two output ports of the coupler, which proves the effectiveness of the RF power-combining network design.

**Figure 12:**
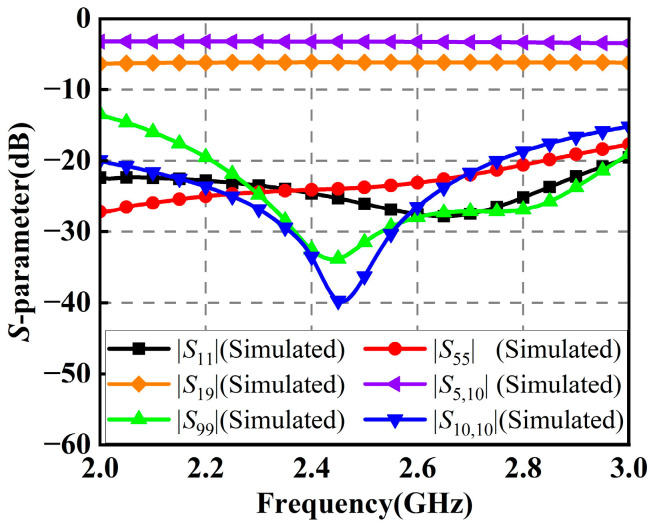
Simulated reflection coefficients and insertion losses of the Wilkinson power dividers.

As shown in Figure 12, the simulated reflection coefficients of the 1-to-2 and 1-to-4 Wilkinson power dividers in the RF power-combining network are both below −20 dB at 2.45 GHz, which means that the Wilkinson power dividers in the network have good impedance matching. Moreover, the insertion loss of the 1-to-4 Wilkinson power divider is 6.1 dB, while the insertion loss of the 1-to-2 Wilkinson power divider is 3.2 dB.

**Figure 13:**
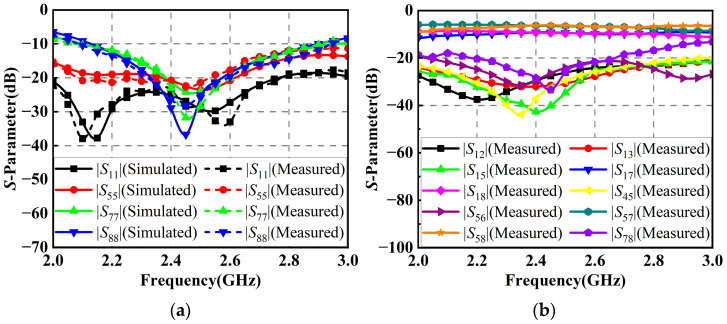
*S*-parameters of the proposed RF power-combining network: (**a**) Reflection coefficients; (**b**) Insertion and isolation performances.

The simulated and measured *S*-parameters of the RF power-combining network are given in Figure 13. As shown in Figure 13a, the simulated and measured reflection coefficients of the network are below −20 dB at the center frequency of 2.45 GHz. In addition, the simulated and measured reflection coefficients within the operating bandwidth of the corresponding antenna array are below −12 dB. As shown in Figure 13b, the measured isolation performances of the network at 2.45 GHz are better than 25 dB. The measured insertion performance from the 1-to-4 Wilkinson power divider’s input port to the coupler’s output port of the network is 9.2 dB, while the one from the input port of the 1-to-2 Wilkinson power divider to the output port of the coupler is 6.4 dB.

## 4. Discussion of the Proposed Rectenna Array

**Figure 14:**
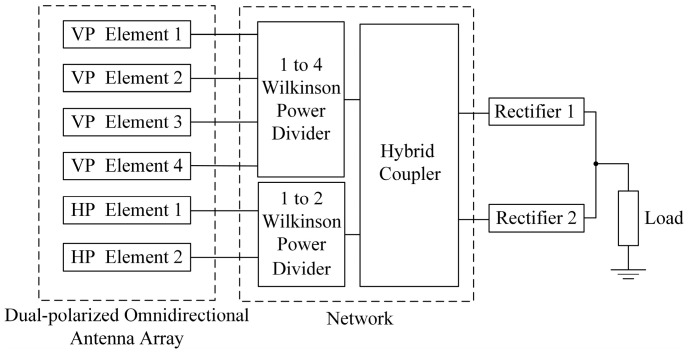
Schematic of the proposed rectenna array.

**Figure 15:**
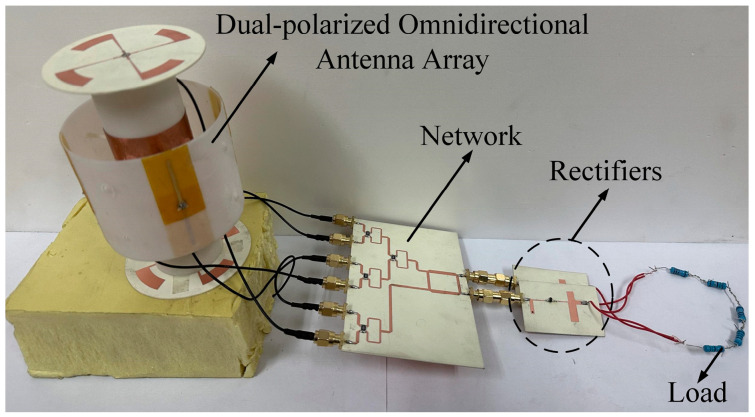
Photograph of the fabricated rectenna array.

The schematic of the proposed rectenna array is shown in Figure 14. The vertically polarized antenna subarray and the horizontally polarized antenna subarray are connected to two Wilkinson power dividers. Then, the RF powers of two different polarizations are fed equally into the two identical rectifiers through a 3-dB hybrid coupler, which finally converge at a resistive load to form the rectenna array. Both rectifiers are the same as the one in Figure 6. The resistance of the resistive load is 950 Ω, which is half the one of a single rectifier. Following the topology in Figure 14, the proposed rectenna array was fabricated. The photograph of the fabricated rectenna array is provided in Figure 15.

**Figure 16:**
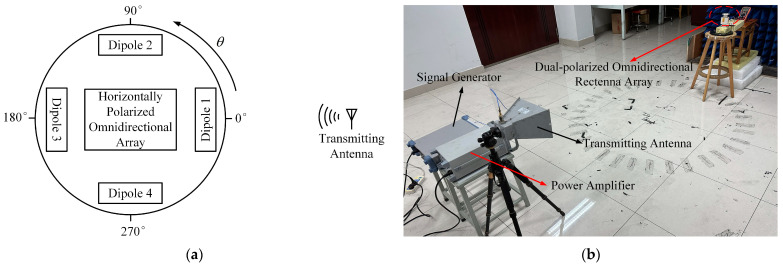
Simulation and test environments of the proposed rectenna array: (**a**) Simulation environment; (**b**) Test environment.

The performances of the proposed rectenna array are simulated and measured under the illumination of EM waves with different incident directions and different polarizations. Figure 16a shows the simulation environment of the rectenna array for harvesting omnidirectional RF energy with different polarizations. The transmitting antenna starts from the horizontal azimuth angle of 0° and turns 15° counterclockwise each time. For the polarization, three cases of horizontal polarization, vertical polarization, and superposition of both horizontal and vertical polarizations are investigated. During the rotation of the transmitting antenna, the number of antenna elements that can receive RF power at each angle is almost always fixed due to the structural symmetry of the rectenna array. In addition, the whole process of omnidirectional RF harvesting can be simplified and analyzed by the cases of incident azimuth angles from 0° and 45°. Using the software Ansys HFSS, power reception under different scenarios was simulated, and the transmission coefficients obtained from the simulation can be used to calculate the power ratio received by each port of the dual-polarized omnidirectional antenna array under different conditions. Then the software ADS is used to co-simulate the RF power-combining network and rectifier array. By adjusting the power ratio of the input source, the PCE in each corresponding state can be obtained.

Figure 16b illustrates the test environment of the proposed rectenna array for RF energy harvesting. During the measurement, an RF signal generator is connected to a power amplifier, and the amplified RF power is transmitted through double-ridge horn antenna points to the rectenna array to be tested. The gain of the horn used in the measurement is 14.5 dBi at 2.45 GHz. The distance between the transmitting horn antenna and the rectenna array is set to 2 m, which satisfies the far-field condition. A standard gain antenna is first placed at the receiving position, and the power density of the incident EM wave at the receiving position can be determined by measuring the received power using a spectrum analyzer. Then the rectenna array is placed on the rotary table at the receiving position. The angle of incidence can be adjusted by controlling the rotary table, and the polarization direction angles of the incident wave can be changed by adjusting the double-ridge horn antenna. A digital multimeter is used to measure the voltage on the load of the rectenna array so that the output DC power can be calculated. During the measurement, when the output power of the amplifier is 32.5 dBm, the corresponding power density at the receiving position is 100 μW/cm^2^. When the output power of the amplifier is increased to 35.5 dBm, the corresponding power density at the receiving position is 200 μW/cm^2^.

The horizontally polarized reception, vertically polarized reception, and both horizontally and vertically polarized reception correspond to the cases when the polarization direction angle is at 0°, 90°, and 45° with the horizontal plane, respectively.

**Figure 17:**
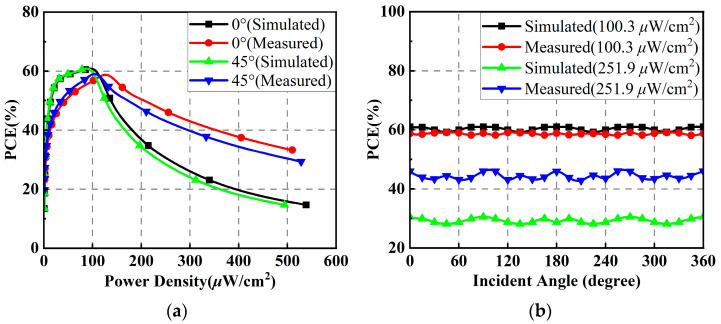
Simulated and measured results of the rectenna array when the polarization direction angle is 0°: (**a**) PCEs versus input power density; (**b**) PCEs versus incident angle.

When the polarization direction angle is 0°, the simulated and measured PCEs of the proposed rectenna array versus the input power density for EM wave incomes at angles *θ* of 0° and 45° are shown in Figure 17a. Under the incidence angles of 0° and 45°, the simulated and measured results are basically the same when the input power density is less than 200 μW/cm^2^. The maximum PCE is about 60%. Figure 17b plots the simulated and measured PCEs of the rectenna array versus the incident angle under input power densities of 100.3 and 251.9 μW/cm^2^. It is not difficult to find that the PCEs of the rectenna array at different power densities are relatively constant with only slight fluctuations when the incidence angle of the electromagnetic wave varies. The main reason why the measured PCEs at higher power densities are better than the simulated ones is that the rectifiers have a wider operating input power range in the measurement.

**Figure 18:**
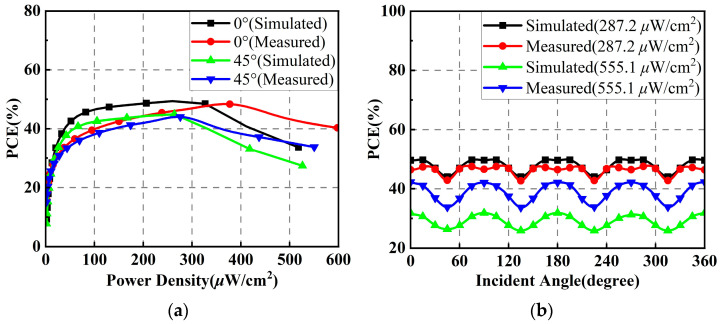
Simulated and measured results of the rectenna array when the polarization direction angle is 90°: (**a**) PCEs versus input power density; (**b**) PCEs versus incident angle.

When the polarization direction angle is 90°, the simulated and measured PCEs of the proposed rectenna array versus the input power density for EM wave incomes at angles *θ* of 0° and 45° are shown in Figure 18a. It can be clearly seen that the PCEs of the rectenna array at the incidence angles of 0° and 45° of the EM waves decrease significantly, and the input power densities corresponding to the peak PCEs are increased. However, the rectenna array is still able to obtain PCEs of more than 40% at input power densities of less than 400 μW/cm^2^. Figure 18b plots the simulated and measured PCEs of the rectenna array versus the incident angle under input power densities of 287.2 μW/cm^2^ and 555.1 μW/cm^2^. It can be found that the fluctuations of PCEs at different power densities increase when the incidence angle of EM waves changes. However, generally, the fluctuations remain within a tolerable range. This could be attributed to the imperfect omnidirectional radiation performance of the antenna array in the vertically polarized state and the fact that the phase superposition may be non-ideal during RF power combining.

**Figure 19:**
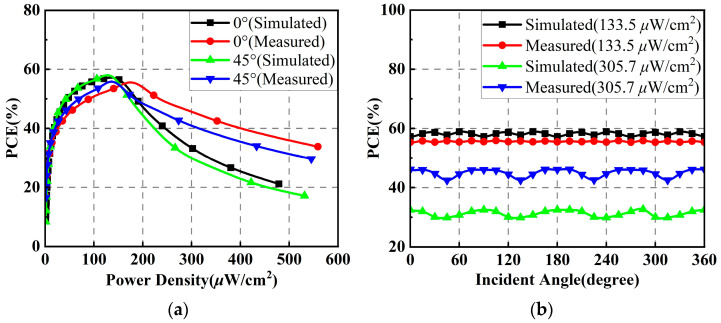
Simulated and measured results of the rectenna array when the polarization direction angle is 45°: (**a**) PCEs versus input power density; (**b**) PCEs versus incident angle.

When the polarization direction angle is 45°, the simulated and measured PCEs of the proposed rectenna array versus the input power density for EM wave incomes at angles *θ* of 0° and 45° are shown in Figure 19a. Similar to the case where the polarization direction angle is 0°, the rectenna array is also capable of achieving maximum PCEs of about 60% at power densities of less than 200 μW/cm^2^, when the incidence angles of the EM wave are 0° and 45°. However, the input power densities under which the peak PCEs are obtained increase slightly. Figure 19b plots the simulated and measured PCEs of the rectenna array versus the incident angle under input power densities of 133.5 μW/cm^2^ and 305.7 μW/cm^2^. Similarly, the PCEs of the rectenna fluctuate slightly at different power densities when the incidence angle of the EM wave varies, demonstrating the stability of the rectenna array for omnidirectional RF energy harvesting.

To describe the performance of the proposed rectenna array more intuitively, the maximum PCEs and their corresponding power densities obtained during simulations and measurements at different polarization direction angles are listed in Table 4. Meanwhile, Table 5 lists the simulated and measured maximum PCEs and PCE fluctuations of the rectenna array at different EM wave incidence angles. According to the data in Table 4 and Table 5, the proposed rectenna array has good omnidirectional RF energy harvesting capability, and the PCE fluctuations in different harvesting scenarios are below 10%.

Table 6 below gives the comparison between the rectenna array in this work and the existing related works. It can be found that the proposed rectenna array can maintain good stability when receiving EM waves with varying incident directions and polarizations. It is competitive with the existing arts in terms of spatial coverage, polarization mode, and PCE fluctuation when used for RF energy harvesting (REH) applications.

## 5. Conclusions

A dual-polarized omnidirectional rectenna array with a hybrid power combining scheme is proposed in this paper. The circularly arranged antenna elements and dipole elements are used to form horizontally and vertically polarized subarrays, respectively. The dual-polarized omnidirectional antenna array is realized by uniting the two subarrays. An RF power-combining network is designed by using the structure of 1-to-2 and 1-to-4 Wilkinson power dividers and a 3-dB hybrid coupler. The designed antenna array, RF power combining network, and rectifiers are fabricated and measured. The impedance matching, isolation performances, and radiation patterns of the antenna array were measured. In addition, the PCEs of the rectifiers and the performances of the RF combining network were measured to verify the validity of the design. Furthermore, the combined dual-polarized omnidirectional rectenna array was also measured. The measured results show that the omnidirectional-recycling capability of the rectenna array can be achieved. The PCEs of the rectenna array in different reception scenarios are over 44% when the input power density is less than 400 μW/cm^2^. On the other hand, the utilization of the RF power combining network enables the rectenna array to maintain excellent stability when receiving EM waves at different incidence and polarization direction angles. Its overall PCE fluctuations are below 10%. Therefore, the proposed rectenna array is suitable for RF energy harvesting applications.

## Figures and Tables

**Table 1:** Optimized dimensions of the proposed antenna array.

Parameter	Value (mm)	Parameter	Value (mm)	Parameter	Value (mm)	Parameter	Value (deg)
*R*_0_	18.1	*d*_1_	0.9	*H*_2_	122	*θ*_1_	42.5
*R*_1_	37	*d*_2_	1.2	*H*_3_	0.8	*θ*_2_	3
*R*_2_	22.6	*d*_3_	0.3	*H*_4_	0.8		
*R*_3_	29.9	*d*_4_	0.7	*w*_a_	6		
*R*_4_	35	*d*_5_	5.6	*w*_b_	1.7		
*R*_5_	2	*d*_6_	0.9	*l*_a_	28.2		
*R*_6_	3	*H*_0_	0.762	*l*_b_	23.2		
*d*_0_	0.3	*H*_1_	60	*l*_c_	2.5		

**Table 2:** Dimensions of the proposed rectifier (unit: mm).

Parameter	Value (mm)	Parameter	Value (mm)	Parameter	Value (mm)	Parameter	Value (mm)
*W*_m1_	1.6	*W*_m5_	4.1	*L*_m2_	10.6	*L*_m6_	7.2
*W*_m2_	0.5	*W*_m6_	2.4	*L*_m3_	6.9	*L*_m7_	11
*W*_m3_	2.1	*W*_m7_	4.8	*L*_m4_	9.7		
*W*_m4_	0.5	*L*_m1_	5	*L*_m5_	6		

**Table 3:** Dimensions of the proposed RF power-combining network (unit: mm).

Parameter	Value (mm)	Parameter	Value (mm)	Parameter	Value (mm)	Parameter	Value (mm)
*w*_0_	1.6	*l*_0_	5	*l*_6_	4.6	*l*_12_	7.5
*w*_1_	0.9	*l*_1_	5.2	*l*_7_	37.6	*l*_13_	5
*w*_2_	73.1	*l*_2_	6	*l*_8_	4	*l*_14_	23.6
*w*_3_	1.6	*l*_3_	16.2	*l*_9_	18.2	*l*_15_	16.7
*w*_4_	2.8	*l*_4_	7.2	*l*_10_	4	*l*_16_	15.4
*w*_5_	1.6	*l*_5_	4	*l*_11_	5	*l*_17_	5.8

**Table 4:** Maximum PCEs and their corresponding power densities at different polarization direction angles.

Polarization Direction Angle	Incident Angle	Maximum PCE for Simulation/Measurement (%)	Power Density Corresponding to Simulation/Measurement (μW/cm^2^)
0°	0°	60.51/58.73	85.33/128.12
	45°	60.51/59.04	78.33/105.35
90°	0°	49.4/48.35	259.62/377.26
	45°	44.9/44.04	263.78/275.83
45°	0°	56.81/55.52	120.27/165.45
	45°	57.8/55.80	123.25/130.75

**Table 5:** Maximum PCEs and PCE fluctuations at different EM wave incidence angles.

Polarization Direction Angle	Power Density (μW/cm^2^)	Maximum PCE for Simulation/Measurement (%)	PCE Fluctuation for Simulation/Measurement (%)
0°	100.3	60.97/59.10	1.73/0.99
	251.9	30.47/45.99	2.37/3.15
90°	287.2	49.73/47.51	5.67/4.82
	555.1	31.82/42.23	5.96/8.55
45°	133.5	58.9/55.92	1.64/0.58
	305.7	32.47/46.12	2.6/3.69

**Table 6:** Comparison between the present design and the existing related arts.

Ref.	Frequency (GHz)	Polarization *	Radiation	Maximum Antenna Gain (dBi)	Maximum PCE (%)	PCE Ripple (%)
[2]	3.75	DLP	Directional	-	34	-
[17]	1.7–2.5	DLP	Directional	5.93	62	-
[28]	3	DLP	Directional	-	20	-
[29]	3.2	LP	Directional	-	40	-
[30]	2.4	LP	Omnidirectional	8.8	38	18
This work	2.45	DLP	Omnidirectional	H-pol: 5.01 V-pol: 1.83	59.04	8.6

* LP and DLP mean linearly polarized and dual linearly polarized, respectively.

## Data Availability

Not applicable.

## References

[1] Popovic Z., Falkenstein E.A., Costinett D., Zane R. (2013). Low-Power Far-Field Wireless Powering for Wireless Sensors. Proc. IEEE.

[2] Dinh M., Ha-Van N., Tung N.T., Le M.T. (2021). Dual-Polarized Wide-Angle Energy Harvester for Self-Powered IoT Devices. IEEE Access.

[3] Fan Y., Liu X., Xu C. (2022). A Broad Dual-Band Implantable Antenna for RF Energy Harvesting and Data Transmitting. Micromachines.

[4] Lin W., Ziolkowski R.W., Huang J. (2019). Electrically Small, Low-Profile, Highly Efficient, Huygens Dipole Rectennas for Wirelessly Powering Internet-of-Things Devices. IEEE Trans. Antennas Propag..

[5] Luo Y., Pu L., Lei L. (2021). Impact of Varying Radio Power Density on Wireless Communications of RF Energy Harvesting Systems. IEEE Trans. Commun..

[6] Shafique K., Khawaja B.A., Khurram M.D., Sibtain S.M., Siddiqui Y., Mustaqim M., Chattha H.T., Yang X. (2018). Energy Harvesting Using a Low-Cost Rectenna for Internet of Things (IoT) Applications. IEEE Access.

[7] Hemour S., Zhao Y., Lorenz C.H.P., Houssameddine D., Gui Y., Hu C.M., Wu K. (2014). Towards Low-Power High-Efficiency RF and Microwave Energy Harvesting. IEEE Trans. Microw. Theory Tech..

[8] Pinuela M., Mitcheson P.D., Lucyszyn S. (2013). Ambient RF Energy Harvesting in Urban and Semi-Urban Environments. IEEE Trans. Microw. Theory Tech..

[9] Xu C., Fan Y., Liu X. (2022). A Circularly Polarized Implantable Rectenna for Microwave Wireless Power Transfer. Micromachines.

[10] Song C., Huang Y., Carter P., Zhou J., Yuan S., Xu Q., Kod M. (2016). A Novel Six-Band Dual CP Rectenna Using Improved Impedance Matching Technique for Ambient RF Energy Harvesting. IEEE Trans. Antennas Propag..

[11] Lin W., Ziolkowski R.W. (2020). Electrically Small Huygens CP Rectenna with a Driven Loop Element Maximizes Its Wireless Power Transfer Efficiency. IEEE Trans. Antennas Propag..

[12] Roy S., Tiang R.J.J., Roslee M.B., Ahmed M.T., Mahmud M.A.P. (2021). Quad-Band Multiport Rectenna for RF Energy Harvesting in Ambient Environment. IEEE Access.

[13] Xie F., Yang G.M., Wen G. (2013). Optimal Design of an Antenna Array for Energy Harvesting. IEEE Antennas Wirel. Propag. Lett..

[14] Hui W., Guo Y., Zhao X. (2023). Polarization Tunable Microstrip Antenna based on Double V-type Metamaterials Cover for Microwave Energy Harvesting. IEEE Antennas Wirel. Propag. Lett..

[15] Wang Y., Zhang J., Su Y., Jiang X., Zhang C., Wang L., Cheng Q. (2022). Efficiency Enhanced Seven-Band Omnidirectional Rectenna for RF Energy Harvesting. IEEE Trans. Antennas Propag..

[16] Qi X., Xu Z., Li H. (2022). High-Efficiency 2-D Multibeam Rectenna Based on Gain Enhanced Patch Array. IEEE Antennas Wirel. Propag. Lett..

[17] Bo S.F., Ou J.H., Dong Y., Dong S.W., Zhang X.Y. (2022). All-Polarized Wideband Rectenna With Enhanced Efficiency Within Wide Input Power and Load Ranges. IEEE Trans. Ind. Electron..

[18] Sakamoto T., Ushijima Y., Nishiyama E., Aikawa M., Toyoda I. (2013). 5.8-GHz Series/Parallel Connected Rectenna Array Using Expandable Differential Rectenna Units. IEEE Trans. Antennas Propag..

[19] Kumar M., Kumar S., Jain S., Sharma A. (2023). A Plug-in Type Integrated Rectenna Cell for Scalable RF Battery Using Wireless Energy Harvesting System. IEEE Microw. Wirel. Technol. Lett..

[20] Chen Y.S., You J.W. (2018). A Scalable and Multidirectional Rectenna System for RF Energy Harvesting. IEEE Trans. Compon. Packag. Manuf. Technol..

[21] Arrawatia M., Baghini M.S., Kumar G. (2016). Broadband Bent Triangular Omnidirectional Antenna for RF Energy Harvesting. IEEE Antennas Wirel. Propag. Lett..

[22] Wagih M., Weddell A.S., Beeby S. (2021). Omnidirectional Dual-Polarized Low-Profile Textile Rectenna with over 50% Efficiency for Sub-μW/cm^2^ Wearable Power Harvesting. IEEE Trans. Antennas Propag..

[23] Zhu G.L., Du J.X., Yang X.X., Zhou Y.G., Gao S. (2019). Dual-Polarized Communication Rectenna Array for Simultaneous Wireless Information and Power Transmission. IEEE Access.

[24] Hu Y.Y., Sun S., Wu H., Yang S., Hu J. (2022). Integrated Coupler-Antenna Design for Multibeam Dual-Polarized Patch-Array Rectenna. IEEE Trans. Antennas Propag..

[25] Zhang H., Gao S.P., Wu W., Guo Y.X. (2018). Uneven-to-Even Power Distribution for Maintaining High Efficiency of Dual-Linearly Polarized Rectenna. IEEE Microw. Wirel. Compon. Lett..

[26] Shen S., Zhang Y., Chiu C.Y., Murch R. (2020). A Triple-Band High-Gain Multibeam Ambient RF Energy Harvesting System Utilizing Hybrid Combining. IEEE Trans. Ind. Electron..

[27] Lee D.J., Lee S.J., Hwang I.J., Lee W.S., Yu J.W. (2017). Hybrid Power Combining Rectenna Array for Wide Incident Angle Coverage in RF Energy Transfer. IEEE Trans. Microw. Theory Tech..

[28] Ashoor A.Z., Ramahi O.M. (2019). Polarization-Independent Cross-Dipole Energy Harvesting Surface. IEEE Trans. Microw. Theory Tech..

[29] Ashoor A.Z., Almoneef T.S., Ramahi O.M. (2018). A Planar Dipole Array Surface for Electromagnetic Energy Harvesting and Wireless Power Transfer. IEEE Trans. Microw. Theory Tech..

[30] Vandelle E., Bui D.H.N., Vuong T.P., Ardila G., Wu K., Hemour S. (2019). Harvesting Ambient RF Energy Efficiently With Optimal Angular Coverage. IEEE Trans. Antennas Propag..

